# How the NaOCl solution concentration and temperature impact chlorine levels, tissue dissolution and pH

**DOI:** 10.1590/0103-644020246279

**Published:** 2025-11-21

**Authors:** Maria Eduarda Paz Dotto, Fernanda Guesser, Julia Menezes Savaris, Camila Segatto Hartmann, Amanda Tavares Germano, Lucas da Fonseca Roberti Garcia, Cleonice da Silveira Teixeira

**Affiliations:** 1 Department of Dentistry, Endodontics Division, Health Sciences Centre, Federal University of Santa Catarina, Florianópolis, SC, Brazil.; 2 Department of Chemistry Analysis Center, Federal University of Santa Catarina, Florianópolis, SC, Brazil.

**Keywords:** Concentration, Free Available Chlorine, Sodium Hypochlorite, Solution pH, Temperature, Tissue Dissolution

## Abstract

This study analyzed the behavior of sodium hypochlorite (NaOCl) at various concentrations and temperatures in relation to free available chlorine (FAC), tissue dissolution (TD), and pH. NaOCl solutions (2.50%, 5.25%, and 8.00% w/v) and distilled water (H_2_O) were evaluated. Each solution was heated to different temperatures (25°C, 37°C, and 60°C) for periods of 5 minutes, 10 minutes, and 15 minutes. For tissue dissolution analysis, bovine tissue samples (n = 10) were immersed in each solution and weighed before and after the designated times. FAC and pH were measured after each heating time. Data normality and homoscedasticity were verified using the Shapiro-Wilk and Levene tests. Solubility data were analyzed using the Kruskal-Wallis test and the Dwass-Steel-Critchlow-Fligner post-hoc test, and pH data were analyzed using one-way ANOVA and the Tukey post-hoc test. FAC data were subjected to a Student's t-test (α = 0.05%). At 25°C, all NaOCl concentrations had similar dissolution effects (p>0.05) and were superior to H_2_O (p<0.05). At 37°C, the dissolution effect of 2.5% NaOCl was similar to water after 5 min (p>0.05). NaOCl at 5.25% and 8.00% dissolved 90-100% of the samples after 10-15 min (37°C and 60°C). FAC in 2.50% NaOCl decreased when heated (37°C and 60°C), and 5.25% NaOCl showed a FAC reduction at 60°C (p<0.05). NaOCl at 8.00% showed similar results at all temperatures (p>0.05). The pH decreased (p<0.05) at 37°C and 60°C. Increasing NaOCl concentration, temperature, and heating time enhanced the tissue dissolution effect. Higher temperatures also reduced the FAC concentration in 2.50% and 5.25% NaOCl, as well as the pH in all evaluated NaOCl concentrations.

## Resumo

Este estudo analisou o comportamento do hipoclorito de sódio (NaOCl) em várias concentrações e temperaturas em relação ao cloro livre disponível (FAC), à dissolução tecidual (TD) e ao pH. Foram avaliadas soluções de NaOCl (2,50%, 5,25%, 8,00% w/v) e água destilada (H2O). Cada solução foi aquecida em diferentes temperaturas (25°C, 37°C, 60°C) pelos períodos de 5 min, 10 min e 15 min. Para a análise da dissolução tecidual, amostras de tecido bovino (n=10) foram imersas em cada solução, sendo pesadas antes e após os tempos designados. O FAC e o pH foram medidos após cada período de aquecimento. A normalidade e a homoscedasticidade dos dados foram verificadas utilizando os testes de Shapiro-Wilk e Levene. Os dados de solubilidade foram analisados pelo teste de Kruskal-Wallis e pelos testes post-hoc de Dwass-Steel-Critchlow-Fligne, enquanto os dados de pH foram analisados por ANOVA de uma via e pelo teste post-hoc de Tukey. Os dados de FAC foram submetidos ao teste t de Student (α=5%). A 25°C, todas as concentrações de NaOCl apresentaram efeitos de dissolução semelhantes (p>0.05) e foram superiores à H2O (p<0.05). A 37°C, o efeito de dissolução do NaOCl a 2,50% foi semelhante ao da água após 5 min (p>0.05). NaOCl a 5,25% e 8.00% dissolveram 90-100% das amostras após 10-15 min (37°C e 60°C). O FAC no NaOCl a 2,50% diminuiu quando aquecido (37°C e 60°C), enquanto o NaOCl a 5,25% mostrou uma redução no FAC a 60°C (p<0.05). O NaOCl a 8,00% apresentou resultados semelhantes em todas as temperaturas (p>0.05). O pH diminuiu (p<0.05) a 37°C e 60°C. O aumento da concentração de NaOCl, da temperatura e do tempo de aquecimento potencializou o efeito de dissolução tecidual. Temperaturas mais elevadas também reduziram a concentração de FAC nas soluções de NaOCl a 2,50% e 5,25%, bem como o pH em todas as concentrações de NaOCl avaliadas.

**Figure:**
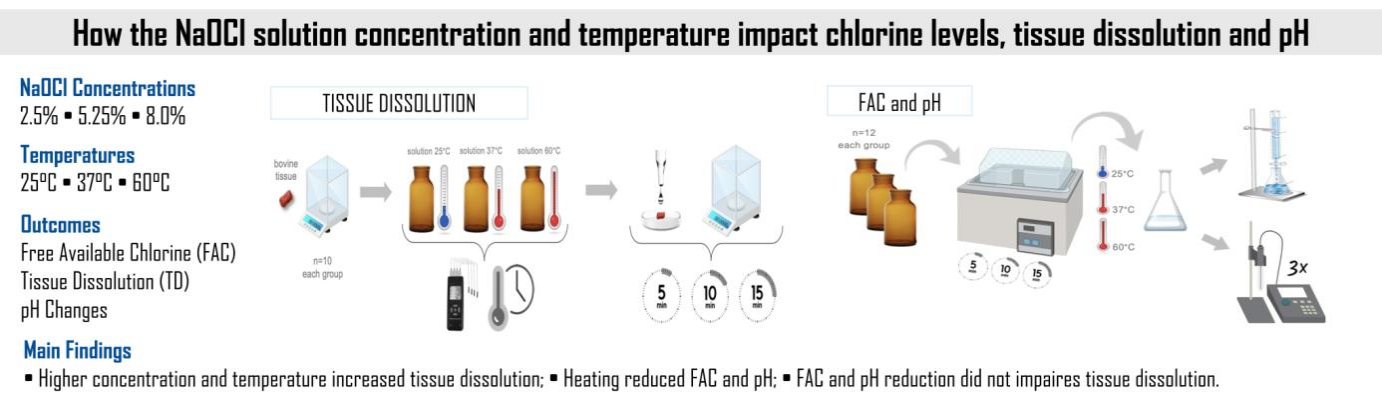

## Introduction

The chemical action of irrigating solutions plays a crucial role in endodontic treatment ^1^. The mechanical action of endodontic instruments alone is insufficient for effectively cleaning, disinfecting, and removing debris from the root canal system, including its anatomical complexities ^1^ ^,^ ^2^.

Above all, due to its tissue-dissolving capability ^3^ ^,^ ^4^ and high antimicrobial power ^5^, sodium hypochlorite (NaOCl) solution is the irrigant of choice in endodontics ^1^ ^,^ ^6^. This solution is obtained through the electrolysis of a concentrated sodium chloride solution ^7^. The properties of NaOCl are intrinsically linked to the formation of hypochlorous acid (HOCl) and hypochlorite ion ( OCl - ), representing the amount of free available chlorine (FAC) in the solution ^7^ ^,^ ^8^. FAC denotes the amount of available chlorine in the solution, and a higher concentration corresponds to faster tissue dissolution ^9^. The hypochlorite ion (OCl^-^) is closely associated with tissue dissolution capacity ^10^, becoming more prominent at elevated pH levels. When the pH of the solution is below 7.4, hypochlorous acid (HOCl) predominates, playing a significant role as a bactericidal agent through the chloramination process ^11^ ^,^ ^12^ ^,^ ^13^. Therefore, pH directly influences the chemical properties of the NaOCl irrigant ^7^ ^,^ ^10^ ^,^ ^11^.

In endodontics, various concentrations of NaOCl are used, ranging from 0.5% to 8.25% ^1^ ^,^ ^8^. The increase in concentration is directly related to enhanced antimicrobial effects and intensified tissue dissolution capacity ^14^ ^,^ ^13^ ^,^ ^16^. However, this increase inversely affects biological compatibility if extruded into periapical tissues ^17^.

Heating the NaOCl solution can enhance its disinfection power and improve smear layer removal ^16^ ^,^ ^18^ ^,^ ^19^ ^,^ ^20^. This process accelerates the reaction rate between solution components, enhances irrigant flow, and facilitates faster organic matter dissolution ^14^ ^,^ ^20^. Furthermore, it promotes greater penetration into dentinal tubules ^9^. However, raising the temperature of the solution can accelerate the reaction between the irrigants, intensifying the depletion of FAC ^21^ ^,^ ^22^. Consequently, this results in a decrease in the properties of the NaOCl solution ^23^. Therefore, the present study aimed to evaluate the behavior of NaOCl solution at different concentrations (2.50%, 5.25%, and 8.00%) and temperatures (25°C, 37°C, and 60°C) in terms of FAC levels, organic tissue dissolution, and pH. The null hypothesis posited that variations in temperature or concentration have no impact on the properties of the tested irrigation solutions.

## Materials and methods

### Tissue dissolution

A sodium hypochlorite (NaOCl) solution at a concentration of 10-12% w/v was obtained and stored in a refrigerated environment at temperatures ranging from 2°C to 8°C, protected from moisture, heat, and direct sunlight, until the beginning of the experiment. The storage time did not exceed 7 days. Different NaOCl concentrations were prepared by diluting with distilled water (H_2_O), resulting in 1 liter of each concentration (2.5%, 5.25%, and 8% w/v) immediately before the experiment.

The sample size was determined based on a previous study ^20^ and calculated using the G*Power 3.1 program (G*Power, 3.1.7, Heinrich Heine Universität Düsseldorf, Germany). To detect a 10% difference, with a statistical power of 80% at a 5% alpha level, the determined sample size was at least nine specimens per group. Therefore, a total of 10 specimens per group was established.

**Figure 1:**
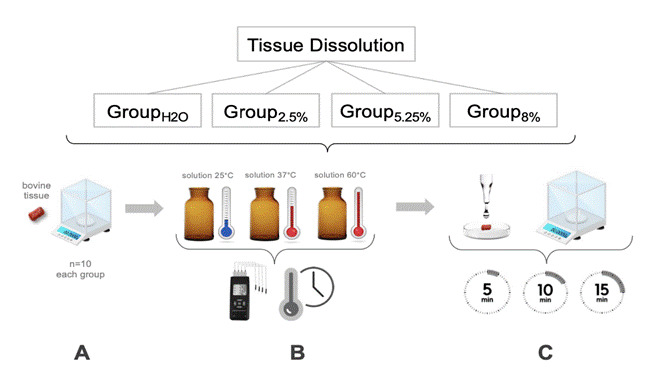
Experimental design flowchart for tissue dissolution analysis. (A) Samples of bovine muscle tissue were cut and weighed on an electronic balance. (B) Solution heated to 25°C, 37°C, and 60°C (reading with type K thermocouple sensor) in a water bath, and samples of bovine tissue were placed in the vials containing 10 mL of each solution according to a pre-defined time. (C) Samples were removed from the solution, washed with water at 25°C, and the weight was again measured. The process was repeated three times (5, 10, and 15 min).

Samples of bovine muscle tissue were cut and weighed on an electronic balance (AW220; Shimadzu, Kyoto, Japan) with an accuracy of 0.1 mg. The sample surfaces were approximately 2 mm wide, 2 mm thick, and 6 mm long, with an approximate weight of 52.0 mg (± 0.002 mg) (Figure 1A). Four groups (G, n=10) were formed according to the evaluated solution as follows:

Group_H2O_ (control): A total of 300 mL of H_2_O was heated to three different temperatures (25°C, 37°C, 60°C). Thus, 30 replicates (10 mL each) were used for each temperature, as follows: At 25°C, 10 bovine tissue samples were initially weighed and then individually immersed in 10 mL of the solution. The specimens were reweighed after 5, 10, and 15 minutes of immersion, with the solution replaced at each interval. Similarly, at 37°C and 60°C, the same number of samples underwent immersion in 10 mL of the solution. Weighing was performed at each time point for all temperatures, with the mean of two repetitions taken, and the solution was renewed at the same intervals.

Group_2.50%_ (2.50% NaOCl): Same protocol as in the Group_H2O_, but with 2.50% NaOCl.

Group_5.25%_ (5.25% NaOCl): Same protocol as in the Group_H2O_, but with 5.25% NaOCl.

Group_8.00%_ (8.00% NaOCl): Same protocol as in the Group_H2O_, but with 8.00% NaOCl.

Amber glass vials with a capacity of 10 mL were used for organizing the groups. Each solution (water and NaOCl at concentrations of 2.50%, 5.25%, and 8.00%) was heated to 25°C, 37°C, and 60°C ± 2°C in a water bath (Ratek Instruments, Boronia, Australia). Temperature readings in °C were taken using a type K thermocouple sensor (GHM Messtechnik, Regenstauf, Germany) in contact only with the irrigant solution. Once the pre-established temperatures were reached, individual samples of bovine tissue were placed in the vials containing 10 mL of each test solution (Figure 1B). After 5 minutes, the samples were removed from the solution, washed for 1 minute with distilled water at 25 °C, gently dried using absorbent paper, and weighed again on the precision balance ^20^. This process was repeated three times to obtain data for immersion periods of 5, 10, and 15 minutes (Figure 1C), with solutions being refreshed between each immersion.

### Free Available Chlorine (FAC) and pH analysis

For the FAC evaluation, the standard iodine/thiosulfate titration method, as described in the British Pharmacopoeia (1973), was employed. To ensure better control of aliquot volumes, NaOCl solutions were prepared through stoichiometric calculation, considering the molar concentration (mol L_-1_) of hypochlorite ion (OCl_-_), the transferred volume from the stock solution (10-12%), and the final volume in the volumetric flask (Table 1).

**Figure 2:**
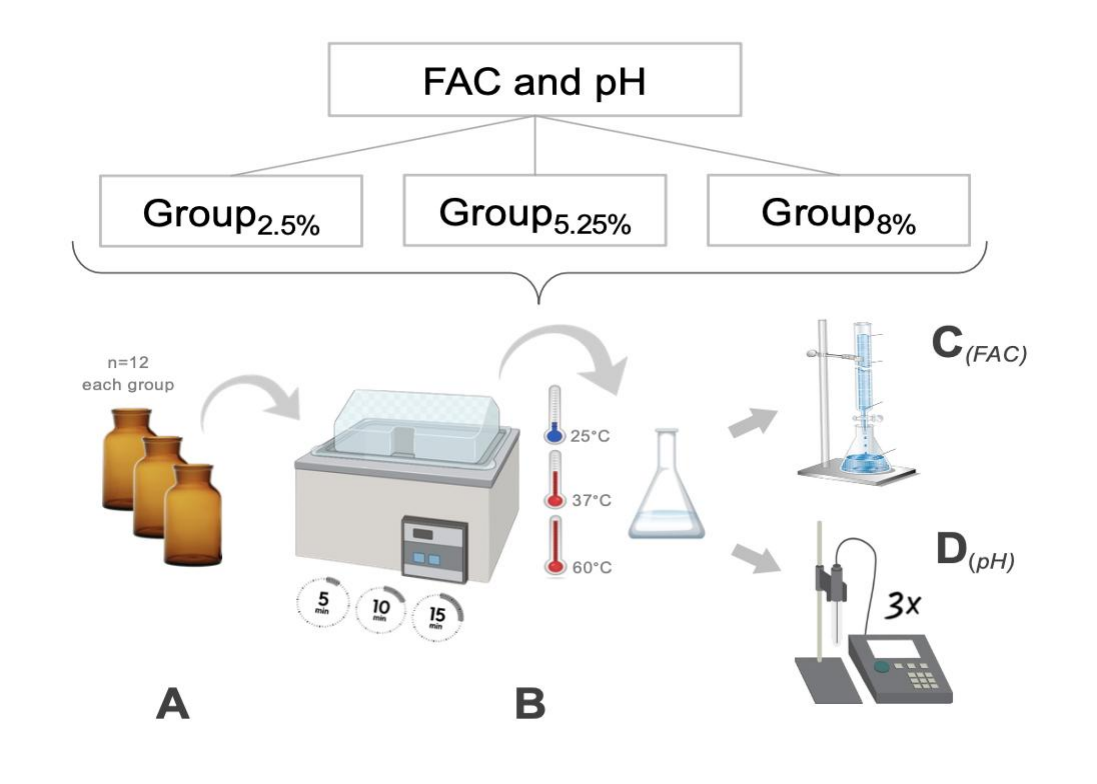
Experimental design flowchart for analysis of FAC and pH in NaOCl solution (A): amber glass vials of 10 mL with a NaOCl solution concentration (2.50%, 5.25%, and 8.00%) separated. (B) The solution was heated to 25 °C, 37 °C, and 60 °C in a water bath for 5, 10, and 15 minutes, respectively. (C) The solution was dispensed into a 25 mL volumetric flask, which was then filled with distilled water to the mark. After homogenization, the titration was performed. (D) Solutions were evaluated in triplicate using a pH meter.

**Table 1:** The molar concentration (mol L^-1^) of hypochlorite ion (M_OCl-_), the transferred volume from the stock solution (10-12%), and the final volume in the volumetric flask.

OCl^-^(%)	M_OCl-_ (mol L^-1^)	Transferred volume (mL)	Final volume (mL)
2.50%	0,330	100	500
5.25%	0,705	205	500
8.00%	1,074	310	500

After preparing the NaOCl solutions immediately before the experiment, 12 amber glass vials with a capacity of 10 mL each were arranged and filled for each group as presented in Figure 2A. Each concentration (2.50%, 5.25%, and 8.00%) of the NaOCl solution was heated to 25 °C, 37 °C, and 60 °C ± 2 °C in a water bath (Ratek Instruments, Boronia, Australia); the temperature reading in °C was conducted using a type K thermocouple sensor (GHM Messtechnik, Regenstauf, Germany), in contact only with the present irrigant solution (Figure 2B). From the amber vials, a 5 mL aliquot of the 2.50% NaOCl solution was drawn and dispensed into a 25 mL volumetric flask, which was then filled with distilled water. After homogenization, a 10 mL volume was transferred with an automatic pipettor to an Erlenmeyer flask, from which titration was performed. The analysis of the 5.25% and 8.00% NaOCl solutions was also conducted using a 5 mL aliquot of their respective prepared solutions, with a 50 mL flask volume. These modifications ensured control in the titration process and allowed stoichiometric estimation, which was considered for the calculation of free available chlorine.

The titration method involves using the volume of consumed thiosulfate (S_2_O_32−_) to determine the amount of chlorine present in the sample. Once the 10 mL of the NaOCl solution is diluted in the Erlenmeyer flask, 25 mL of H_2_O, 20 mL of acetic acid (CH_3_COOH), and 1 gram of Potassium Iodide (KI) are added. The mixture is gently homogenized, and titration is initiated: the Erlenmeyer flask is positioned under a burette filled with thiosulfate solution (S_2_O_32−_), which is added to the sample until a yellowish color is obtained. At this point, six drops of starch are added, causing the sample to turn a dark blue color. Titration proceeds slowly until the endpoint is reached, which is identified by the disappearance of the blue color. The volume of consumed thiosulfate in the burette is noted. To estimate the available chlorine content in the sample, the following reaction ^3^ occurring in the Erlenmeyer flask is considered:

$${OCl}_{\left(aq\right)}^{-}+3I^{-}+2H_{\left(aq\right)}^{+}\;\to \;I_{3\;\left(aq\right)}^{-}+{Cl}_{\left(aq\right)}^{-}+H_{2}O_{\left(l\right)}$$

$$I_{3\;\left(aq\right)}^{-}+2\;S_{2\;}O_{3(aq)}^{2-}\to \;3I_{\left(aq\right)}^{-}+\;S_{4}O_{6(aq)}^{2-}$$

Therefore, for stoichiometry, it is considered that 1 mole of hypochlorite ion (OCl_-_) reacts with 2 moles of thiosulfate. Since, during dilution, the quantity of solute does not change, the calculation of the number of moles (n) follows the equation.n=M ×V, where M represents the molar concentration (mol L_-1_) and V is the volume of the solution.

With the known molar concentration of the thiosulfate solution produced in the laboratory, and the manipulated volume of OCl_-_, along with the titration providing the volume of consumed thiosulfate, it is possible to determine the molar concentration of the hypochlorite ion (OCl_-_), analyzed as FAC content. The free available chlorine was measured at 0 minutes, after 5 minutes, 10 minutes, and 15 minutes of heating at temperatures of 25°C, 37°C, and 60°C, with two replicates at each time interval, totaling 24 titrations for each concentration (2.50%, 5.25%, and 8.00%) (Figure 2C). The pH of the solutions was evaluated in triplicate during the same period using a pH meter (Figure 2D) (Micronal, PH-1700, São Paulo, Brazil).

### Statistical analysis

The data were tabulated using Microsoft Excel and analyzed with the Jamovi software (Jamovi®, Statistical for Windows, V.2.2.5). Initially, the data were explored for normality and homoscedasticity using the Shapiro-Wilk and Levene tests, respectively. For the dissolution test data, due to the non-normal distribution among the groups, the Kruskal-Wallis test was applied, followed by the Dwass-Steel-Critchlow-Fligner multiple comparisons post-hoc test for detailed differences. The pH data, which were normally distributed, were evaluated using one-way ANOVA and Tukey's post-hoc test. The iodometric titration data, which were normally distributed and paired, were analyzed using the Student's t-test. The significance level was set at 5%.

## Results

### Tissue dissolution

The initial weight of each bovine tissue sample evaluated in the dissolution test was standardized at 0.52 mg (± 0.002), with no statistically significant difference between the groups (p > 0.05) (Table 2).

At 25°C, no variation in tissue weight was observed in the G_H2O_ group (p>0.05) relative to the initial weight, regardless of immersion time. At 37°C and 60°C, a significant reduction in tissue weight was observed after 5, 10, and 15 minutes of immersion in H_2_O, compared to its initial weight (p < 0.05). However, no significant difference was noted between the 10- and 15-minute time periods (p < 0.05).

**Table 2:** Dissolution of the bovine tissue (weight in grams), according to the solution concentration, temperature, and time of action.

Temperature	Solution	Time of action			
		0 min	5 min	10 min	15 min
25°C	H_2_O	0.052 (0.003)^Aa^	0.049 (0.004)^Aa^	0.049 (0.005)^Aa^	0.048 (0.004)^Aa^
	2.50%NaOCl	0.053 (0.002)^Aa^	0.036 (0.003)^Bb^	0.020 (0.005)^Bc^	0.009 (0.003)^Bd^
	5.25%NaOCl	0.050 (0.002)^Aa^	0.032 (0.06)^Bb^	0.016 (0.004)^Bc^	0.003 (0.003)^Bd^
	8.00%NaOCl	0.050 (0.002)^Aa^	0.018 (0.003)^Cb^	0.003 (0.001)^Cc^	0.0001 (0.000)^Cd^
37°C	H_2_O	0.053 (0.003)^Aa^	0.041 (0.003)^Ab^	0.035 (0.002)^Ac^	0.034 (0.002)^Ac^
	2.50% NaOCl	0.053 (0.002)^Aa^	0.035 (0.006)^Ab^	0.017 (0.004)^Bc^	0.005 (0.003)^Bd^
	5.25% NaOCl	0.052 (0.002)^Aa^	0.023 (0.004)^Bb^	0.004 (0.002)^Cc^	0.001 (0.001)^Cd^
	8.00% NaOCl	0.052 (0.002)^Aa^	0.009 (0.006)^Cb^	0.001 (0.001)^Dc^	0.000 (0.000)^Cd^
60°C	H_2_O	0.052 (0.002)^Aa^	0.040 (0.003)^Ab^	0.036 (0.002)^Ac^	0.033 (0.002)^Ac^
	2.50% NaOCl	0.052 (0.002)^Aa^	0.029 (0.002)^Bb^	0.011 (0.002)^Bc^	0.004 (0.002)^Bd^
	5.25% NaOCl	0.052 (0.002)^Aa^	0.013 (0.004)^Db^	0.002 (0.000)^Cc^	0.000 (0.000)^Cd^
	8.00% NaOCl	0.053 (0.003)^Aa^	0.020 (0.002)^Cb^	0.004 (0.002)^Cc^	0.000 (0.000)^Cd^

Different uppercase superscript letters represent a significant difference between the solutions at each temperature and action time (columns) (p<0.05).

Different lowercase superscript letters represent a significant difference between action times for each solution (lines) (p<0.05)

**Figure 3:**
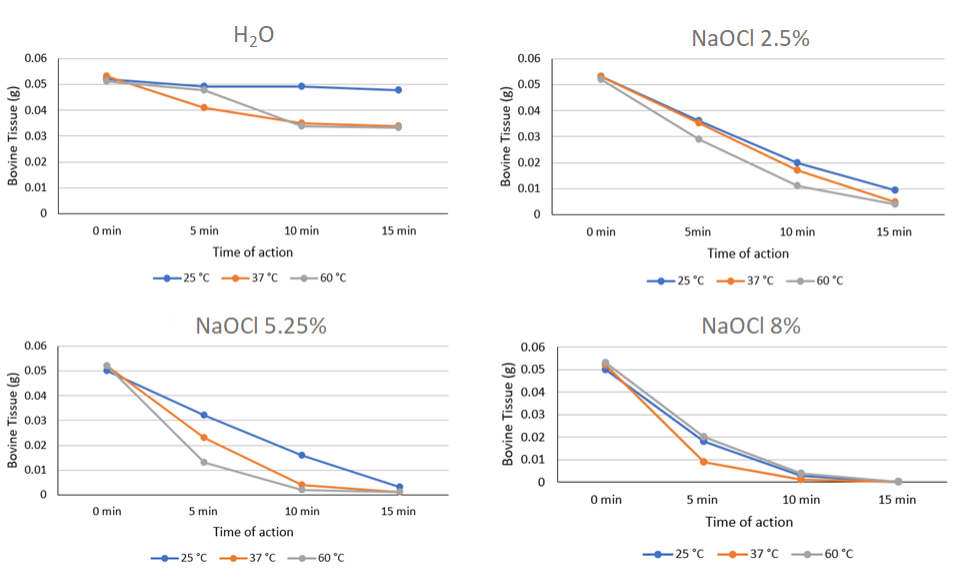
Line graphs depicting the variation in bovine tissue mass (measured in grams) over different immersion times and irrigating solutions utilized.

Immersing the bovine samples in different concentrations of NaOCl (2.5%, 5.25%, 8%) and at various temperatures (25°C, 37°C, 60°C) resulted in significant weight loss across all evaluated time periods (p < 0.05) (Figure 3).

When the solutions were compared with each other within each action time and temperature, it was observed that at 25°C, after 5, 10, and 15 minutes of immersion, the dissolution promoted by 2.50% NaOCl was similar to that promoted by 5.25% NaOCl (p > 0.05). Both were higher (p<0.05) than water but lower (p<0.05) than 8.00% NaOCl. At 37 °C, the dissolution of 2.50% NaOCl was similar (p>0.05) to water after 5 min of immersion. At other times and concentrations, NaOCl had a greater dissolving effect than water (p<0.05). Both 5.25% and 8.00% NaOCl promoted dissolution between 90-100% of the samples after 10 and 15 min of immersion at 37°C and 60°C.

### FAC and pH analysis

The results obtained from FAC and pH analysis are presented in Table 3. For 2.50% NaOCl, a statistical difference was found only between 37°C and 60°C (p<0.05). For 5.25% NaOCl, the temperatures of 25°C and 60°C differed from each other (p<0.05), showing a decline in the average FAC. However, at 37°C, the results were similar (p>0.05) to the other temperatures. The FAC for 8.00% NaOCl yielded similar results across all temperatures (p > 0.05).

Within each concentration, a statistically significant difference (p<0.05) was found between all temperatures (25°C ≠ , 37°C ≠ , and 60°C), with a significant reduction in average pH values ​​when the solutions were heated to 37°C and 60°C (p<0.05).

**Table 3:** Mean and standard deviation (in parentheses) of FAC and pH in different NaOCl concentrations and temperatures.

Concentration	Temperature	FAC	pH
		Mean (SD)	Mean (SD)
2.50%	25°C	2.40 (0.037)^AB^	12.8 (0.012)^A^
	37°C	2.42 (0.023)^A^	12.3 (0.236)^B^
	60°C	2.36 (0.051)^B^	11.6 (0.063)^C^
5.25%	25°C	4.97 (0.014)^A^	13.0 (0.069)^A^
	37°C	4.95 (0.022)^AB^	12.4 (0.050)^B^
	60°C	4.94 (0.029)^B^	11.7 (0.140)^C^
8.00%	25°C	7.51 (0.019)^A^	12.9 (0.252)^A^
	37°C	7.55 (0.041)^A^	12.6 (0.077)^B^
	60°C	7.54 (0.052)^A^	11.6 (0.052)^C^

Different superscript letters indicate FAC and pH values ​​that are statistically different from each other, within each group/concentration (1-way Anova and Tukey test, p<0.05).

## Discussion

The properties of NaOCl solutions can be modified by altering their temperature, pH, or concentration ^14^ ^,^ ^23^. However, there is no consensus in the literature regarding the ideal characteristics that fully optimize all properties of this solution. Heating a solution increases the kinetic energy of the molecules present, resulting in reduced viscosity, cohesive forces, and surface tension ^24^. In this context, studies suggest that heating a NaOCl solution is a feasible strategy to enhance its action ^14^ ^,^ ^18^ ^,^ ^20^.

Based on this study, it was observed that increasing the temperature and concentration of the solutions influenced FAC levels, promoted greater tissue dissolution, and induced changes in the average pH values, thus rejecting the initially formulated null hypothesis.

The tissue dissolution capacity is one of the crucial properties linking the use of NaOCl to the success of endodontic treatment ^1^ ^,^ ^4^. According to our findings, the synergistic action of increased concentration, coupled with higher temperatures, enhances tissue dissolution. The organic tissue dissolution by NaOCl is primarily attributed to its ability to break down proteins into smaller peptides and amino acids ^7^. The hypochlorite anion (OCl⁻) reacts with the amide groups in proteins, leading to their denaturation and eventual dissolution ^10^. The key reaction involves the oxidation of sulfhydryl groups (-SH) in cysteine residues, disrupting disulfide bonds and compromising the structural integrity of proteins ^12^. This oxidation is further facilitated by the generation of chloramines during the interaction between hypochlorous acid (HOCl) and amino acids, enhancing protein breakdown ^13^. The increase in temperature enhanced the ability of NaOCl to dissolve organic material, even at lower concentrations ^14^. Greater molecular agitation promotes increased contact between the molecules and the material to be dissolved, while also reducing surface tension and enhancing the wettability of the surrounding structures ^16^ ^,^ ^20^. Previous studies have reported similar findings ^14^ ^,^ ^16^ ^,^ ^19^ ^,^ ^20^, and some have shown that the dissolution speed is similar at higher concentrations and lower temperatures, such as 5.00% NaOCl at 20°C, compared to lower concentrations at higher temperatures, as observed in 1% NaOCl at 36°C ^14^ ^,^ ^19^. Additionally, 5.00% NaOCl at 60°C dissolved a greater amount of organic tissue within 5, 10, or 15 minutes compared to 5.00% NaOCl at 48°C ^20^. Our results demonstrated that NaOCl concentrations of 5.25% and 8.00% were effective in dissolving 90-100% of sample weight after 10 and 15 minutes of immersion at 37°C and 60°C, consistent with findings by Christensen et al. ^23^. In a similar study, it was observed that bovine pulp immersed in 5.00% NaOCl for 5, 15, and 30 minutes showed significantly greater dissolution at 15 minutes compared to 5 minutes ^25^. However, it is important to note that, even though the increased temperature of the NaOCl solution accelerates the dissolution rate of bovine pulp, this dissolution reaches a plateau at temperatures between 60-75°C ^26^.

The bovine tissue samples used in this study provided a practical analysis of organic dissolution, serving as a comparative model to human dental pulp by accurately simulating its volume and weight ^20^. The preference for this type of tissue in other research ^20^ ^,^ ^26^ is based on its advantages in standardizing the size and weight of the samples ^14^. The time intervals and temperatures used in this methodology were selected for their clinical relevance and were based on previous studies ^20^ ^,^ ^21^.

The dissolution power of 2.5% NaOCl resembled that of water at 37°C after 5 minutes. Although distilled water is considered an inert control solution in terms of tissue dissolution capacity, it is essential to acknowledge that increased temperature can alter its physicochemical properties, such as reduced viscosity and increased molecular mobility ^24^. When heated, its kinetic energy increases, which may promote the destabilization of cellular structures, hydration of macromolecules, and the onset of protein degradation through physical processes such as thermal denaturation ^27^. This highlights the importance of the thermal factor as a potential adjuvant in the action of irrigants, even those with little or no specific chemical activity ^16^. However, after 10 and 15 minutes, 2.5% NaOCl surpassed water in its dissolving effect. This suggests that even a lower NaOCl concentration, when renewed and kept in prolonged contact with organic matter, improves tissue dissolution ^20^.

In endodontic clinical practice, the constant renewal of the preheated solution also ensures the renewal of the FAC to react with organic tissue ^28^. During intracanal irrigation, the human body can quickly regulate the temperature of NaOCl, potentially reducing the benefits of heating ^16^. This rapid decrease in solution temperature makes selecting higher concentrations an appealing option during endodontic treatment, as it ensures greater availability of available chlorine and, consequently, enhances dissolving and antimicrobial action, particularly in cases of necrotic pulp ^1^ ^,^ ^5^ ^,^ ^8^ ^,^ ^19^ ^,^ ^29^. However, higher concentrations also have greater cytotoxic potential and detrimental effects on dentin structure and periapical tissues ^17^. Therefore, the choice of NaOCl concentration should balance the biocompatibility of lower concentrations with the efficiency of more concentrated solutions ^7^. Although the temperature of preheated solutions drops after intracanal irrigation, they react more rapidly with substrates while their temperature remains elevated ^30^. Consequently, renewing preheated solutions may be a valuable strategy to maximize the effectiveness of less concentrated solutions ^31^.

The reaction kinetics of NaOCl are temperature-dependent ^10^. Heating NaOCl solutions increases reaction rates, including decomposition into chlorate and oxygen ^13^. The FAC, representing the oxidizing capacity expressed as the amount of chlorine ^23^, was evaluated in this study. The results indicated that the FAC values of the tested solutions (2.40%, 5.00%, and 7.50%) were slightly below the initially considered concentrations (2.50%, 5.25%, and 8.00%). It is known that FAC decreases more rapidly at higher temperatures, after prolonged agitation, and at higher concentrations ^21^. This study observed the most significant discrepancies between the initially considered concentration and FAC values in NaOCl 8.00%, followed by NaOCl 5.25%. However, these differences were below 10%, staying within acceptable limits ^29^.

It is well established that FAC levels decrease more rapidly at higher temperatures, with prolonged agitation, and in more concentrated solutions ^21^. Although our results showed statistically significant FAC reductions under certain heating conditions, previous studies indicate that such losses do not necessarily compromise clinical effectiveness ^23^ ^,^ ^31^. Heating enhances the tissue dissolution and antimicrobial activity of NaOCl by improving penetration, reducing viscosity, and accelerating reaction kinetics ^20^ ^,^ ^23^ ^,^ ^31^. Moreover, other studies have reported minimal FAC degradation in NaOCl solutions at various concentrations, even after extended heating ^19^ ^,^ ^20^ ^,^ ^31^.

The antimicrobial efficacy of NaOCl is also highly dependent on pH ^11^. At higher pH levels, OCl⁻ predominates, while at lower pH levels, HOCl becomes the dominant compound ^10^. HOCl is a more potent antimicrobial agent due to its neutral charge, enabling it to penetrate microbial cell walls more effectively ^13^. However, maintaining a high pH ensures the stability of NaOCl solutions and reduces their decomposition rates ^7^. The dual influence of pH on efficacy and stability necessitates careful control in clinical practice to optimize antimicrobial action without compromising solution longevity ^31^.

Given the initially recorded pH between 11 and 12, it was observed that the free available chlorine in this study was primarily associated with the hypochlorite ion (OCl^-^) concentration in the solution. It is known that a higher pH, approaching 12, increases the tissue dissolution power of the solution ^30^. However, although heating caused statistical differences in pH values within each group, it was not sufficient to induce clinically significant changes in pH during the analyzed time periods ^26^.

Within the limitations identified in this study, the experimental setup stands out, highlighting the complexity of maintaining or achieving a specific temperature in all samples. Standardizing solution volume, bovine tissue sample, and bath temperature was challenging, as it aimed to provide the highest possible reliability in the results. In this regard, more in vitro and in vivo research is suggested to assess the clinical relevance of the results obtained in the present study.

## Conclusion

Based on the current data, increasing the NaOCl concentration, temperature, and heating time resulted in a greater tissue dissolution power of the solutions. The increase in temperature also reduced the free available chlorine at 2.5% and 5.25% NaOCl, as well as the pH at all concentrations evaluated.
